# The role of macrophage ion channels in the progression of atherosclerosis

**DOI:** 10.3389/fimmu.2023.1225178

**Published:** 2023-07-31

**Authors:** Xin Wu, Sidhant Singla, Jianhua J. Liu, Liang Hong

**Affiliations:** ^1^ Department of Medicine, University of Illinois at Chicago, Chicago, IL, United States; ^2^ Department of Pathology, University of Illinois at Chicago, Chicago, IL, United States; ^3^ Department of Physiology and Biophysics, University of Illinois at Chicago, Chicago, IL, United States

**Keywords:** macrophage, atherosclerosis, ion channel, foam cell formation, inflammatory disease

## Abstract

Atherosclerosis is a complex inflammatory disease that affects the arteries and can lead to severe complications such as heart attack and stroke. Macrophages, a type of immune cell, play a crucial role in atherosclerosis initiation and progression. Emerging studies revealed that ion channels regulate macrophage activation, polarization, phagocytosis, and cytokine secretion. Moreover, macrophage ion channel dysfunction is implicated in macrophage-derived foam cell formation and atherogenesis. In this context, exploring the regulatory role of ion channels in macrophage function and their impacts on the progression of atherosclerosis emerges as a promising avenue for research. Studies in the field will provide insights into novel therapeutic targets for the treatment of atherosclerosis.

## 1 Introduction

Atherosclerosis is a complex process that involves the interaction between oxidized low-density lipoproteins (oxLDLs), macrophages, endothelial cells, and vascular smooth muscle cells (1). It is an inflammatory disease of the large artery driven by macrophage activation and infiltration. Through scavenger receptors, macrophages uptake oxLDLs and other lipids, leading to foam cell formation and fatty streak lesions characteristic of early atherosclerosis. The macrophage-derived foam cells frequently undergo apoptosis to give rise to cholesterol-rich necrotic cores to advance atherosclerotic lesions (2).

Ion channels, transmembrane proteins that allow the flow of ions across cell membranes, are emerging as important regulators of macrophage function (3, 4). Recent studies have revealed diverse roles of ion channels in macrophages and their implications in immune responses and inflammatory diseases, including atherosclerosis (5). In the present review, we discuss ion channel regulation of macrophage function and summarize studies in macrophage ion channel dysfunctions associated with pathogenesis of atherosclerosis.

## 2 Macrophage in the progression of atherosclerosis

Macrophages play a crucial role in the initiation, progression, and complications of atherosclerosis (2, 6, 7). In the presence of risk factors such as high blood pressure, dyslipidemia, diabetes, and smoking, plasma lipoproteins enter the intima by permeating through the vessel wall, where they are modified into oxidized lipoproteins including oxLDLs (8–11). This leads to the activation of endothelial cells, triggering the expression of adhesion and chemotactic factors that attract monocytes. Consequently, monocytes differentiate into macrophages and transform into foam cells in the intima.

The foam cells are formed by the uptake and accumulation of oxLDLs through scavenger receptors in macrophages, leading to the development of fatty streak lesions and atherosclerotic plaques (7). Macrophages express several scavenger receptors including SR-A1 (Scavenger Receptor - Class A1) and CD36 (i.e., SR-B2, Scavenger Receptor - Class B2). The SR-A1 binds to the modified LDL such as oxLDL and is involved in the subendothelial translocation of LDL. SR-A1 function is associated with JNK2 signal pathway, it was reported that JNK2-dependent phosphorylation of SR-A1 promotes the uptake of LDL in macrophage and enhances the foam cell formation (12). The CD36 is the predominant scavenger receptor for oxLDL, and the binding between CD36 and oxLDL triggers TLRs signaling pathway to promote pro-inflammatory responses to mediate the progression of foam cell formation and atherosclerosis (13).

Meanwhile, macrophages in atherosclerotic lesions produce various inflammatory cytokines such as IL-1β, IL-6, IL-12, IL-18, and TNF to promote smooth muscle cell proliferation and recruit other inflammatory cells (14). For example, IL-1β induces angiogenesis through the recruitment of myeloid and endothelial cells. IL-6 promotes activation of endothelial cells and induces smooth muscle cells proliferation. IL-12 stimulates the differentiation of T cells and recruits T cells into the atherosclerotic plaques. IL-18 enhances the expression of scavenger receptor CD36 to accelerate atherosclerosis. And TNF induces expression of adhesion molecules and enhances the recruitment of other inflammatory cytokines into the plaques. Taken together, these sustained inflammatory responses create an atherogenic environment, further regulating plaque progression (2).

Within the microenvironment of atherosclerotic plaques, macrophages receive various stimulation and polarize into distinct subtypes. The macrophage subtypes, including M1 and M2, play diverse roles in the advancement of atherosclerosis. The M1 macrophages secrete pro-inflammatory cytokines, contribute to plaque instability, and promote thrombosis. In contrast, M2 macrophages exhibit anti-inflammatory properties, promote tissue repair, and aid in the resolution of inflammation. Imbalances in macrophage polarization affect plaque stabilization in the development of atherosclerosis (6).

## 3 Ion channels in macrophages

Ion channels are transmembrane proteins that enable the passage of ions across cell membranes and regulate physiological processes (15–23). Recent studies have shown ion channels regulate macrophage function (3, 4). Several ion channels in macrophages are implicated in immune responses and inflammatory diseases. The macrophage ion channels include potassium channel, transient receptor potential (TRP) channel, calcium channel, mechanosensitive Piezo channel, chloride channel, and proton channel (3–5).

Potassium channels are crucial regulators of macrophage membrane potential and ion homeostasis. Macrophages express a variety of potassium channels, including inward-rectifying potassium channels (K_ir_), voltage-gated potassium channels (K_v_), and Ca^2+^-activated potassium channels (K_Ca_) (5, 24). These channels modulate macrophage membrane potential, calcium signaling, cytokine release, and phagocytosis. Dysregulation of potassium channels can lead to abnormal macrophage activation and cause chronic inflammation (25, 26).

TRP channels are a diverse family of cation channels that play important roles in various physiological and pathological processes (27). Several TRP channels, such as TRPA1, TRPC3, TRPM2, and TRPV4, are expressed in macrophages (5). Emerging evidence suggests their involvement in macrophage function and inflammatory responses. TRP channels have been shown to modulate macrophage M1/M2 polarization and play roles in calcium homeostasis and reactive oxygen species (ROS) production. Some TRP channels have been implicated in the formation of macrophage-derived foam cells and the development of atherosclerosis (28–34).

Macrophages also express multiple types of calcium channels, including store-operated calcium channels (SOCCs) and voltage-gated calcium channels (Ca_v_) (3, 5). Calcium ions (Ca^2+^) serve as universal second messengers and play a central role in macrophage signaling. Calcium influx through the calcium channels triggers intracellular signaling cascades, leading to macrophage activation, phagocytosis, and cytokine secretion. Abnormal calcium signaling in macrophages has been associated with various inflammatory diseases (35, 36).

In addition, macrophages express chloride channels involved in cellular volume regulation and mechanosensitive Piezo1 that mediated cellular mechano-signaling. Macrophages also express proton channels regulating the activation of phagocyte NADPH oxidase to regulate the process of phagocytosis (3, 5).

## 4 Macrophage ion channels associated with atherosclerosis

In the development of atherosclerosis, macrophage ion channels play important roles in several key cellular events, such as macrophage polarization and infiltration, cell proliferation and migration, and foam cell apoptosis. Recent studies indicated that macrophage ion channels are associated with the pathogenesis of atherosclerosis (**Table 1**).

**Table 1:** The role of macrophage ion channels in atherosclerosis.

Ion channel	Gene	Roles in atherosclerosis	Study model of atherosclerosis	Reference
K_Ca_3.1	*KCNN4*	K_Ca_3.1contributes to atherogenesis in mice and humans.	*Apoe^-/-^ mice*	Toyama et al. (37),
		Blocking K_Ca_3.1 suppresses plaque instability by inhibiting macrophage polarization toward an M1 phenotype.	*Apoe^-/-^ mice*	Xu et al. (25),
		Blockade of macrophage K_Ca_3.1 inhibits cellular oxLDL accumulation and decreases proinflammation factors expression via STAT3/CD36 axis.	*Apoe^-/-^ mice*	Jiang et al. (38),
K_v_1.3	*KCNA3*	Blockade of K_v_1.3 prevents plaque formation.	*Rats*	Wu et al. (39),
		Kv1.3 is a potential binding partner of preImplantation factor (40) and regulates PIF–mediated atherosclerosis.	*Apoe^-/-^ mice*	Chen et al. (40),
		Mediates macrophage migration in atherosclerosis by regulating ERK activity.	*In vitro*	Kan et al. (26),
		K_v_1.3 regulates connexin37-mediated atherosclerosis.	*Apoe^-/-^ mice*	Liao et al. (41),
K_ATP_ (Kir6.1/6.2)	*KCNJ8, KCNJ11*	Atherosclerosis impairs relaxation of the carotid artery in response to activation of K_ATP_ channel.	*Monkeys*	Faraci et al. (42)
		K_ATP_/ERK1/2 pathway is implicated in macrophage-derived foam cell formation.	*In vitro*	Zhao et al. (43)
		K_ATP_ mutants are risk factors for atherosclerosis.	*Population study*	Chatterjee et al. (44)
K_ir_2.1	*KCNJ2*	K_ir_2.1 regulates lipid uptake and foam cell formation through modulating the expression of scavenger receptors.	*In vitro*	Zhang et al. (45)
TRPA1	*TRPA1*	Activation of TRPA1 protects against atherosclerosis.	*Trpa1^-/-^Apoe^-/-^ mice*	Zhao et al. (30)
		TRPA1 regulates macrophages phenotype plasticity, deletion of TRPA1 increases atherosclerosis plaques.	*Trpa1^-/-^Apoe^-/-^ mice*	Wang et al. (29)
TPRC1	*TPRC1*	TPRC1 is highly expressed in macrophage-rich atheroma areas.	*Pigs*	Li et al. (46)
TRPC3	*TRPC3*	Deficiency of TRPC3 reduces early lesion burden and necrotic core of advanced plaques; TRPC3-deficient macrophages polarized to the M1 phenotype show reduced apoptosis.	*Trpc3^-/-^Apoe^-/-^ mice*	Tano et al. (47)
		Overexpression of the TRPC3 increases atherosclerotic lesions.	*TgESTrpc3Apoe^-/-^ mice*	Smedlund et al. (48), Smedlund et al. (49)
		Deficiency of TRPC3 in macrophages reduces necrosis and content of M1 macrophages in atherosclerotic plaques.	*LysM^cre^Trpc3^flox/flox^Ldlr^-/-^ mice*	Solanki et al. (32)
		Deficiency of TRPC3 in macrophages reduces calcification and osteogenic features in advanced atherosclerotic plaques.	*LysM^cre^Trpc3^flox/flox^Ldlr^-/-^ mice*	Dube et al. (31)
		The miR-26a alleviates the development of atherosclerosis by regulating TRPC3.	*Apoe^-/-^ mice*	Feng et al. (50)
TRPM2	*TRPM2*	TRPM2 enhances vascular reactivity during development of atherosclerosis.	*Apoe^-/-^ mice*	Dai et al. (51)
		TRPM2 deletions protects against atherosclerosis by suppresses the activation of the CD36 signaling.	*Trpm2^-/-^Apoe^-/-^ mice; Cd11b^cre^Trpm2^flox/flox^ Apoe^-/-^ mice*	Zong et al. (28)
		TRPM2 contributes to the progression of hypercholesterolemia-induced atherosclerosis.	*Trpm2^-/-^Apoe^-/-^ mice*	Zhang et al. (52)
TRPV4	*TRPV4*	Activation of TRPV4 inhibits monocyte adhesion and atherosclerosis.	*Apoe^-/-^ mice*	Xu et al. (53)
		TRPV4 regulates oxLDL induced macrophage foam cell formation.	*Trpv4^-/-^ mice*	Goswami et al. (34); Gupta et al. (33)
		Inhibition of TRPV4 by ginkgetin abrogates JNK2 activation, inflammation in macrophages, and macrophage foam cell formation.	*C57BL/6 wild type mice*	Alharbi et al. (54)
Piezo1	*Piezo1*	Regulation of inflammatory response, and macrophage migration.	*LysM^cre^Piezo1^flox/flox^ Ldlr^-/-^ mice*	Pan et al. (55)
VRCC	*LRRC8A*	VRCC regulates macrophage-derived foam cell formation and atherosclerosis.	*Apoe^-/-^ mice*	Hong et al. (56)
SOCC	*Orai1*	Inhibition of Orai1 SOCC attenuates the development of atherosclerosis.	*Apoe^-/-^ mice*	Liang et al. (36)
Na_v_1.4, Na_v_1.9	*SCN4A, SCN11A*	Inhibition of Na_v_1.4/1.9 reduces atherosclerosis by suppressing macrophage proliferation.	*Apoe^-/-^ mice*	Sun et al. (57)

### 4.1 Calcium-activated potassium channel (K_Ca_)

The K_Ca_3.1 is the predominant subtype of calcium-activated potassium channels in macrophages. K_Ca_3.1 regulates macrophage activity and plays an essential role in the progression of atherosclerosis.

One study found that K_Ca_3.1 expression is upregulated in macrophages within atherosclerotic plaques in *Apoe^-/-^* mouse model of atherosclerosis and human patients. Inhibition of K_Ca_3.1 with the treatment of TRAM-34 and clotrimazole prevents macrophage activation (37). Moreover, the migratory response of K_Ca_3.1^-/-^ macrophages was significantly reduced than in K_Ca_3.1^+/+^ macrophages, indicating a role of K_Ca_3.1 in the activation of macrophages during the atherosclerosis progression. Xu et al. showed that K_Ca_3.1 regulates macrophage polarization. Blocking K_Ca_3.1 suppresses macrophage polarization towards the M1 phenotype, reducing atherosclerotic plaque instability (25). Moreover, a recent study discovered that K_Ca_3.1 modifies the development of atherosclerosis via the STAT3/CD36 signaling axis (38).

### 4.2 Voltage dependent potassium channel (K_v_)

K_v_1.3 is one of the voltage-gated potassium channels predominantly expressed in macrophages. K_v_1.3 regulates the membrane potential of immune cells and is an important modulator of calcium signaling and cytokine production. In macrophages, K_v_1.3 regulates the activation and proliferation of the cells, as well as the production of pro-inflammatory cytokines such as tumor necrosis factor-alpha (TNF-α) and interleukin-1 beta (IL-1β).

Previous studies showed that pharmacological inhibition of K_v_1.3 channels reduces atherosclerotic lesion area in mouse models (39), suggesting a pro-atherosclerotic role of K_v_1.3. The mechanisms by which K_v_1.3 underlies the development of atherosclerosis are associated with preimplantation factor (58) and extracellular signal-regulated kinase (ERK) signaling pathway. K_v_1.3 is proposed to be a binding partner of PIF and regulates PIF-mediated atherosclerosis (40). K_v_1.3 has also been shown to modify ERK activity to promote macrophage migration during the progression of atherosclerosis. Inhibition of K_v_1.3 channel attenuates macrophage migration and reduces the phosphorylation level of ERK1/2 (26). Additionally, other studies reported that connexin is involved in K_v_1.3-mediated atherosclerosis (41).

### 4.3 Inward-rectifying potassium channel (K_ir_)

The primary macrophage K_ir_ involved in atherosclerosis is ATP-sensitive potassium channels (K_ir_6.1/6.2, or K_ATP_). In macrophages, the K_ATP_ channel is essential for the regulation of inflammation and the immune response.

The first study showing the relationship between K_ATP_ channel and atherosclerosis is from a monkey model of atherosclerosis, and it was shown that atherosclerosis impairs the relaxation of the carotid artery in response to activation of the K_ATP_ channel (42). Further studies revealed that K_ATP_ participates in macrophage-derived foam cell formation. Zhao et al. found that the downregulations of total cholesterol and esterified cholesterol concentrations induced by hydrogen sulfide (H_2_S), were reversed by K_ATP_ blocker glibenclamide, suggesting that K_ATP_ channel promotes the formation of macrophage-derived foam cells (43). Moreover, a population study indicated that K_ATP_ mutants are risk factors for atherosclerosis (44).

In addition to K_ATP_, another inward-rectifying potassium channel K_ir_2.1 also participates in the development of atherosclerosis. K_ir_2.1 regulates macrophage maturation and differentiation and plays a crucial role in lipid uptake and foam cell formation by modulating the expression of scavenger receptors (45).

### 4.4 Transient receptor potential ankyrin channel (TRPA)

The transient receptor potential ankyrin 1 (TRPA1) channel is a non-selective cation channel widely expressed in immune cells, including macrophages. TRPA1 channels play a key role in regulating inflammation. In recent years, several studies have explored the potential role of TRPA1 channels in atherosclerosis.

TRPA1 has been shown to regulate the cholesterol metabolism of macrophage-derived foam cells. OxLDL-induced lipid accumulation of macrophages is exacerbated by either inhibition or loss of function of TRPA1, leading to the progression of atherosclerotic plaques. On the other hand, treatment with TRPA1 agonists alleviates the development of atherosclerosis in *Apoe^-/-^* mice, indicating that TRPA1 protects against atherosclerosis (30). A recent study found that TRPA1 modifies macrophage phenotype plasticity. Inhibition of TRPA1 enhances M1 marker genes expression whereas downregulates M2 genes expression (29).

### 4.5 Transient receptor potential canonical channel (TRPC)

TPRC3 is one of the primary TRPC channels expressed in macrophages. In atherosclerosis, macrophage TRPC3 channel activation enhances inflammation and the development of atherosclerotic plaques. TRPC3 channel activation in macrophages can increase the expression of inflammatory cytokines and chemokines, promote the recruitment of additional immune cells to the site of inflammation, and contribute to the formation of atherosclerotic lesions.

A bone marrow transplantation study revealed that macrophage deficiency of TRPC3 reduces early lesion burden and necrotic core of advanced plaques in *Apoe^-/-^* mice (47). Moreover, macrophage-specific deletion of TRPC3 was reported to decrease necrosis and content of apoptotic M1 macrophages in advanced atherosclerotic plaques of mice (31, 32). The miR-26a was shown to alleviate the development of atherosclerosis by regulating TRPC3 (50). Additionally, studies in endothelial cells discovered that endothelial overexpression of the human TRPC3 channel increased the size and cellularity of advanced atherosclerotic lesions in mice model of atherosclerosis (48, 49).

In addition to TRPC3, another TRPC channel TRPC1 has been shown to be predominantly expressed in macrophage-rich atheroma areas, indicating macrophage TRPC1 plays a role in atherogenesis (46). The mechanisms by which TRPC1 channels regulate macrophage function in atherosclerosis are not fully understood. Further research is required to elucidate the signaling pathways and molecular mechanisms involved in TRPC1-mediated atherosclerosis.

### 4.6 Transient receptor potential melastatin channel (TRPM)

Transient receptor potential melastatin channel member 2 (TRPM2) is highly expressed in macrophages and promotes atherosclerotic progression (28, 51, 52). It was shown that both global and macrophage-specific TRPM2 deletions could protect *Apoe^-/-^* mice against atherosclerosis (28, 52). Inhibition of TRPM2 channel activity in macrophages decreases the production of ROS and pro-inflammatory cytokines, and reduces the size of atherosclerotic lesions in multiple mice models of atherosclerosis (28, 52). TRPM2 deficiency in macrophages decreases the uptake of oxLDL, and reduces macrophage infiltration, foam cell formation, and inflammatory responses. Further studies showed that TRPM2 activation is required for CD36-induced oxLDL uptake and macrophage inflammatory responses. Deletion of the TRPM2 gene or inhibiting TRPM2 channel activity suppresses the activation of the CD36 signaling, suggesting that the TRPM2–CD36 axis plays a vital role in atherogenesis (28).

### 4.7 Transient receptor potential vanilloid channel (TRPV)

Transient receptor potential vanilloid channel member 4 (TRPV4) has been implicated in the formation of macrophage-derived foam cells and the development of atherosclerosis.

TRPV4 is expressed and functional in mouse macrophages. It is required for oxLDL-induced macrophage foam cell formation and regulates the uptake of oxLDL (33, 34). Inhibition of TRPV4 by ginkgetin abrogates JNK2 activation, inflammation in macrophages, and macrophage foam cell formation (54). These results indicate that TRPV4 activity is essential for macrophage foam cell formation and atherosclerosis progression. In addition to its direct effects on macrophages, TRPV4 contributes to atherosclerosis by regulating endothelial cell function. In a study using human monocytes, inhibition of TRPV4 reduces monocyte/macrophage adhesion to endothelial cells to regulate the progression of atherosclerosis (53).

### 4.8 Other ion channels

In addition to potassium channels and TRP channels, several other macrophage ion channels were implicated in atherosclerosis.

Recent studies have revealed that Piezo type mechanosensitive ion channel 1 (Piezo1) in macrophage has been implicated in atherosclerosis (55, 59, 60). This channel appears to play a pro-atherosclerotic role in atherogenesis. It was shown that macrophage specific deletion of *Piezo1* gene significantly reduced atherosclerotic plaques in *Ldlr^-/-^* mice model of atherosclerosis (55). Piezo1 was proposed to participate in macrophage inflammatory activation, proliferation, and migration/infiltration to mediate the progression of atherosclerosis (59, 60).

Additionally, macrophage chloride channels have been linked with atherosclerosis (56). It was reported that volume-regulated chloride channel (VRCC) plays an essential role in macrophage foam cell formation (56), and the activity of VRCC was enhanced in *Apoe^-/-^* mice model of atherosclerosis. The activation of VRCC accelerated the formation of macrophage foam cells, whereas the chloride blockers inhibition of VRCC impaired the foam cell formation (56).

Moreover, macrophage calcium and sodium channels are also linked with atherogenesis. It was shown that Orai1 store-operated calcium channel (SOCC) is required for oxLDL-induced Ca^2+^ influx in macrophages. And *in vivo* studies revealed that inhibition of Orai1 SOCC attenuates the development of atherosclerosis (36). In addition, inhibition of voltage-gated sodium channel (Na_v_) suppressed macrophage proliferation and reduced atherosclerotic lesions in the *Apoe^-/-^* mouse model, shedding light on the role of Na_v_ sodium channels in atherogenesis (57).

## 5 Conclusions and perspectives

We discuss ion channels regulation of macrophage function during the progression of atherosclerosis, as well as summarize recent studies in macrophage ion channel families associated with atherosclerosis. Macrophages are critical players in the pathogenesis of atherosclerosis, influencing multiple stages of plaque development and plaque stability. Ion channels play critical roles in macrophage biology, regulating diverse cellular processes that impact macrophage activation, polarization, phagocytosis, and cytokine secretion. Dysregulation of ion channels is implicated in macrophage-mediated atherogenesis, making them attractive targets for therapeutic intervention.

**Figure 1:**
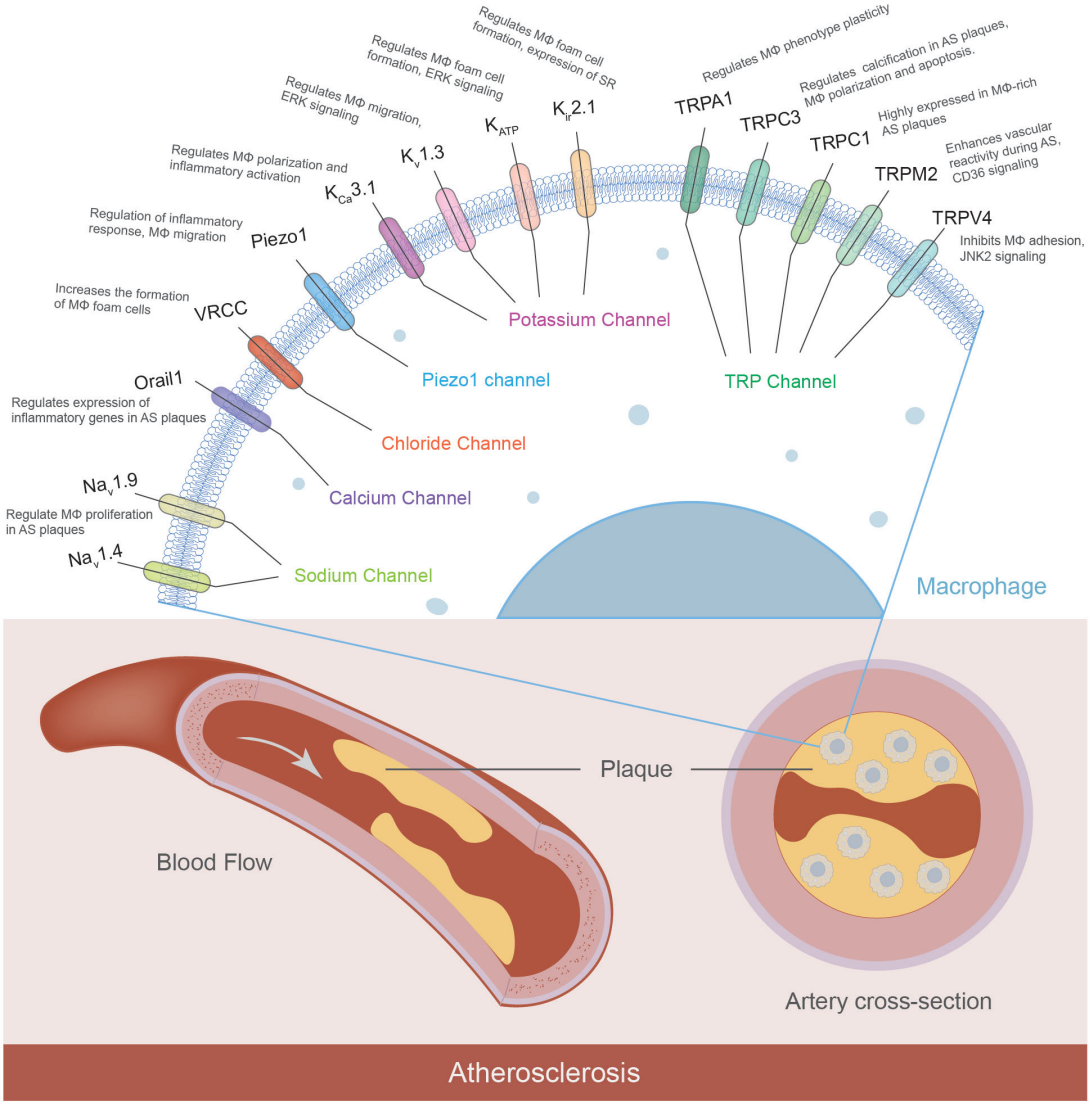
Macrophage ion channels in atherosclerosis. Macrophages express a variety of ion channels, and some of them have been characterized to contribute to the pathophysiology of atherosclerosis. The macrophage ion channels implicated in atherogenesis include K^+^ channels (K_Ca_3.1, K_v_1.3, K_ATP_, K_ir_2.1), TRP channels (TRPA1, TRPC1, TRPC3, TRPM2, TRPV4), Orai1 Ca^2+^ channel, volume-regulated Cl^-^ channel VRCC, mechanosensitive Piezo1, and voltage-gated Na^+^ channels Na_v_. (MΦ: macrophages; AS: atherosclerosis; SR: scavenger receptor).

Although significant progress was made in the functional characterization of the K^+^, TRP, Ca^2+^, and Cl^-^ channels in the progression of atherosclerosis (**Figure 1**), emerging evidence indicated other macrophage ion channels, such as intracellular channels and H^+^ channels, are potentially novel targets against atherosclerosis.

For example, studies have shown that ryanodine receptor 3 (RyR3) channel mutations are associated with atherosclerosis in populations (61). RyR3 is one of the ryanodine receptor channel isoforms expressed on the endoplasmic reticulum of immune cells, including macrophages (62, 63), RyR3 mutations may cause the dysfunction of the channel, which in turn, lead to abnormal calcium signaling linked with the development of atherosclerosis.

Meanwhile, accumulated evidence suggests that H_v_1 proton channel is associated with atherosclerosis. H_v_1 channel controls acid extrusion from cells and regulates cellular pH homeostasis (64). It is highly expressed in macrophages, and its activity promotes macrophage migration and inflammatory cytokines secretion (65, 66). The microarray data has revealed that H_v_1 was remarkably upregulated during atherogenesis and downregulated along with the atherosclerotic lesion regression (67), indicating that H_v_1 is linked with atherogenesis and involved in the pathological process of this disease.

Moreover, acid-sensing ion channel member 1 (ASIC1) was recently proposed to play a role in atherosclerotic development. ASIC1 channel in macrophages decreases ATP-binding cassette transporter A1 (ABCA1)-mediated cholesterol efflux, indicating a role of macrophage ASIC1 in lipid metabolism and atherosclerosis progression (68).

Future studies are required to characterize new roles of these channels in the pathogenesis of atherosclerosis. Meanwhile, studies have shown that ion channels regulate genetic expression in various cells (69–72), it remains to determine if ion channels regulate the gene transcriptional networks controlling macrophage activation linked with atherogenesis. The investigation in this field will extend our understanding of the function of macrophage ion channels in human diseases and discover novel targets against atherosclerosis.

## Author contributions

LH conceptualized the review. XW created the Figure and Table. XW, SS, JL, and LH wrote and edited the manuscript. All authors contributed to the article and approved the submitted version.
